# The Effects of Larval Nutrition on Reproductive Performance in a Food-Limited Adult Environment

**DOI:** 10.1371/journal.pone.0017399

**Published:** 2011-03-30

**Authors:** Caitlin Dmitriew, Locke Rowe

**Affiliations:** 1 Institute of Evolutionary Biology and Environmental Sciences, University of Zürich, Zürich, Switzerland; 2 Department of Ecology and Evolutionary Biology, University of Toronto, Toronto, Canada; Michigan State University, United States of America

## Abstract

It is often assumed that larval food stress reduces lifetime fitness regardless of the conditions subsequently faced by adults. However, according to the environment-matching hypothesis, a plastic developmental response to poor nutrition results in an adult phenotype that is better adapted to restricted food conditions than one having developed in high food conditions. Such a strategy might evolve when current conditions are a reliable predictor of future conditions. To test this hypothesis, we assessed the effects of larval food conditions (low, improving and high food) on reproductive fitness in both low and high food adults environments. Contrary to this hypothesis, we found no evidence that food restriction in larval ladybird beetles produced adults that were better suited to continuing food stress. In fact, reproductive rate was invariably lower in females that were reared at low food, regardless of whether adults were well fed or food stressed. Juveniles that encountered improving conditions during the larval stage compensated for delayed growth by accelerating subsequent growth, and thus showed no evidence of a reduced reproductive rate. However, these same individuals lost more mass during the period of starvation in adults, which indicates that accelerated growth results in an increased risk of starvation during subsequent periods of food stress.

## Introduction

The conditions experienced during early development may have strong downstream effects on the adult phenotype, and therefore on fitness. When juvenile nutritional conditions are good, animals tend to mature at a larger body size and earlier age [1]. Both traits are strongly correlated with fitness in a range of species [2]–[6]. These data suggest a “silver spoon” scenario, in which favourable juvenile growth conditions leads to higher adult fitness. In contrast, the environment-matching hypothesis posits that this silver spoon effect depends on a continuation of favourable conditions into the adult stage [7]. For example, the adult phenotype produced by nutritional stress during the juvenile stage, rather than being simply a smaller-scale copy of the phenotype produced by high food conditions, may actually perform better than the latter in similarly food poor adult environments.

The hypothesis that developmental plasticity matches the phenotype to its future environment has its roots in the study of metabolic syndromes. In humans and other mammals, food stress early in life appears to produce a “thrifty” phenotype that is better suited to continued food stress than to conditions of high food abundance [8]–[10]. This phenotype results from permanent metabolic changes, induced in juveniles, that affect how ingested energy is converted into reserves. Although this increased efficiency is advantageous in energy-limited environments, in energy-abundant environments these individuals suffer from a higher incidence of diabetes, obesity and other metabolic disorders [11]–[13].

It is also known that nutritional stress during growth affects relative allocation to components of the adult phenotype, so that both body composition and allometric relationships are affected [6], [14]–[21]. These observations raise the possibility that allocation decisions are shaped by natural selection to match the adult phenotype to the expected adult environment. For example, the costs of maintenance and locomotion are generally lower for small individuals [22]. Other traits characteristic of food restriction, such as reduced wing loading and higher fat content [23]–[25], also contribute to fitness by improving dispersal ability, and thus the ability to escape poor local conditions and to survive subsequent periods of food restriction [26]–[27]. According to this scenario, it is possible that the adult phenotype produced by restricted larval diets perform better than the phenotype produced by *ad libitum* larval feeding when subsequently facing food poor conditions as adults. Yet, only a few studies tracking the fitness consequences of juvenile food stress beyond the transition to the adult stage do so under stressful adult conditions, so the environment-matching hypothesis has rarely been tested [28]–[30].

We use the ladybird beetle, *Harmonia axyridis*, to test the environment-matching hypothesis. Periods of food restriction during larval development of this beetle results in an adult beetle that is smaller and that has greater fat stores, on average, than those reared in non-limiting conditions [31]–[32]. When food conditions improve before pupation occurs, the beetles may compensate for overall body size and mass, but body composition may still be affected. Both larval and adult *H. axyridis* feed primarily on aphids, populations of which may be ephemeral. As a result, it is predicted that this species should demonstrate highly plastic growth and reproductive strategies in response to food availability. Indeed, studies have shown that both larval and adult beetles are capable of surviving prolonged food stress, though growth and reproductive performance are adversely affected relative to beetles provided with aphids *ad libitum* [31], [33]. Given the short pupation time in this species (4–5 days), larval food levels are likely to be predictive of the conditions into which the adult ecloses. In this experiment, we test whether the phenotypes induced by larval food stress reflect an adaptive matching of the adult phenotype to its expected environment, or whether the “silver spoon” of resource abundance during the larval stage invariably leads to higher adult fitness regardless of the conditions experienced by adults.

## Methods

### (a) General methods

A 3×2 factorial design was used to test the effects of juvenile and adult resource conditions and their interactions on fitness. Larvae were raised in one of three larval environments: high (*H*), low (*L*) and improving (*I*). The latter treatment simulates a local environment in which aphid populations begin to increase before the end of the larval growth period. These individuals may be the most poorly equipped for food stress during the adult stage because they typically undergo costly compensatory growth acceleration [31], perhaps in anticipation of improving conditions. Newly eclosed females were then allocated to one of two adult treatments (high or low) simulating abundant local nutrition or nutritional stress for adults, and the effects of the interaction between larval and adult diet on post-eclosion weight gain/loss, reproductive fitness and survival were assessed. According to the environment-matching hypothesis it was predicted that larvae reared at low food would have higher reproductive success and survival than high food larvae when nutritionally stressed as adults.

Adult ladybird beetles were collected from a natural population in Renfrew, Ontario in April 2009. These beetles were fed aphids (*Acyrthosiphon pisum*) *ad libitum*. Larvae hatching from these eggs on May 11 and 12 were placed individually in 15 cm diameter Petri dishes and assigned randomly to one of the three larval food treatments (N = 120 larvae for each of the three treatments). Larvae in the low food treatment were fed every second day throughout larval development. Larvae in the “improving” treatment were fed every second day until day 8, after which they were fed daily. In the high food treatment, larvae were fed daily from hatch to eclosion (a more detailed description of rations is provided in [32]).

Newly eclosed adults from the three treatments were weighed, sexed, and pronotum width measured within 24–48 hours of eclosion and prior to feeding of adults. This allowed the sclera to harden, preventing damage due to handling. Males were frozen for measurements, and females were placed individually in 500 ml clear plastic tubs and were randomly assigned to one of the two adult treatments. In the low adult food treatment (N = 48, 46, and 44 for High, Improving and Low larval treatments, respectively) water but no food was provided until day eight post eclosion. This treatment simulated eclosion into a period of poor conditions, These conditions simulated a strong food stress for eclosing ladybird beetles; in addition to lack of local prey, adult beetles must also pay energetic costs of searching for more productive patches. In the high adult food treatment (N = 57, 50 and 52 for High, Improving and Low larval treatments, respectively), adults were fed 10 aphids daily during this period and were provided with a cotton ball soaked in sugar water (15 g organic cane sugar/100 ml water). On day eight, adults were re-weighed to assess weight gain or loss during this period in each treatment. After this point, both treatments received a 10-aphid daily ration and sugar water.

### (b) Female reproductive success and longevity

In order to assess treatment effects on reproductive performance and its relationship to larval and adult food conditions, we measured latency to first mating, latency to first oviposition and total reproductive output over the first 28 days post-eclosion. Randomly selected males from a stock population were paired with females daily starting at 2 pm on day 4 post-eclosion. If no copulation occurred within 1 hour, a new male was placed in the container for a second hour. If no copulation occurred within this time period, we assumed that the female was not receptive to mating. This was repeated each day until mating occurred, and this date of first copulation was noted. To assess time to oviposition and fecundity, eggs from each female were counted daily until day 28 post-eclosion. *H. axyridis* are reported to live for 30–90 days under laboratory conditions [33]. A previous experiment showed that the highest rate of egg production occurred in the first two weeks of oviposition under conditions equivalent to the high adult food treatment used here. Our study period was chosen to encompass this period while avoiding high mortality in adult beetles and the decline in hatching success that commences at about 2 months of age in this species.

## Results

### (a) Survival and growth

**Figure 1:**
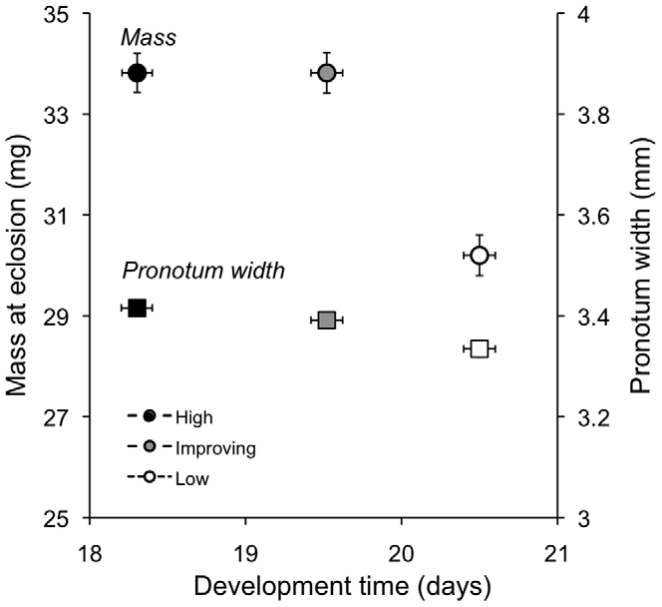
Mass (circles) and pronotum width (square symbols) at eclosion for each larval feeding treatment. Error bars represent 1 SE from the mean and if not visible are smaller than the symbols.

A total of 297 adults eclosed (degrees of freedom reported are reduced for some analyses due to damage or malformation in traits of interest). We found no significant difference in probability of surviving to eclosion among larval treatments (Χ^2^ = 2.14, d.f. = 2, *P* = 0.34), though there was slightly lower mortality among high food larvae (13%) compared to the improving and low conditions (both 20%). As expected, low food larvae eclosed both smaller and lighter than larvae reared at high food. Although larval size at day eight in the improving treatment was noticeably reduced compared to the high food treatment [31], acceleration of growth rate during the latter half development caused these larvae to compensate fully for mass and pronotum width by the time of eclosion (Figure 1; Mass: F_2, 291_ = 39.6, *P*<0.0001, pronotum width: F_2, 286_ = 10.1, *P*<0.0001; Tukey's *post-hoc* test: *H* = *I*>*L* for both traits). Males were on average smaller than females (Mass: F_1, 291_ = 114.6, *P*<0.0001, pronotum width: F_1, 286_ = 41.3, *P*<0.0001) and there was no interaction between sex and larval treatment for either measure of body size (*P*>0.3 for both traits).

**Figure 2:**
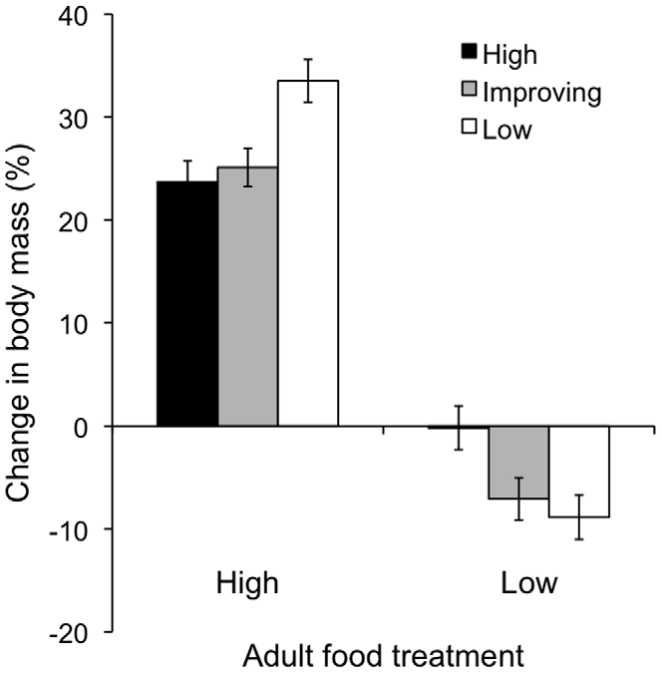
Mass gained or lost (as percentage of initial mass) in response to adult feeding treatment for first week after eclosion. As sexes did not differ, data from males and females are combined. Error bars one standard error from the mean.

Mass gained or lost during the first week as adults depended upon the larval rearing conditions (Table 1, Figure 2). More mass was gained in the high adult food treatment than at low food, regardless of larval diet. When adult food was restricted, those reared on low or improving larval diets lost considerably more mass than those reared at high food, both relative to initial size (Table 1) and in absolute terms (data not shown; *P* = 0.002). There was no effect of treatment on adult female survival during the four weeks of the experiment (larval treatment: X^2^ = 0.40, d.f. = 1, *P*>0.3; adult treatment: X^2^ = 0.086, d.f. = 1, *P*>0.7).

**Table 1:** Effect of larval conditions (high, improving or low food) on the amount of mass gained or lost during the first week as adults (fed or starved).

Source	df	SS	F	P
Larval trt	2	0.06	1.46	0.23
Adult trt	1	7.44	384.24	<0.0001
Larval * Adult trt	2	0.40	10.33	<0.0001
Error	218			
Total	286			

### (b) Effect of larval and adult food levels on time to reproductive maturity and egg production in females

**Figure 3:**
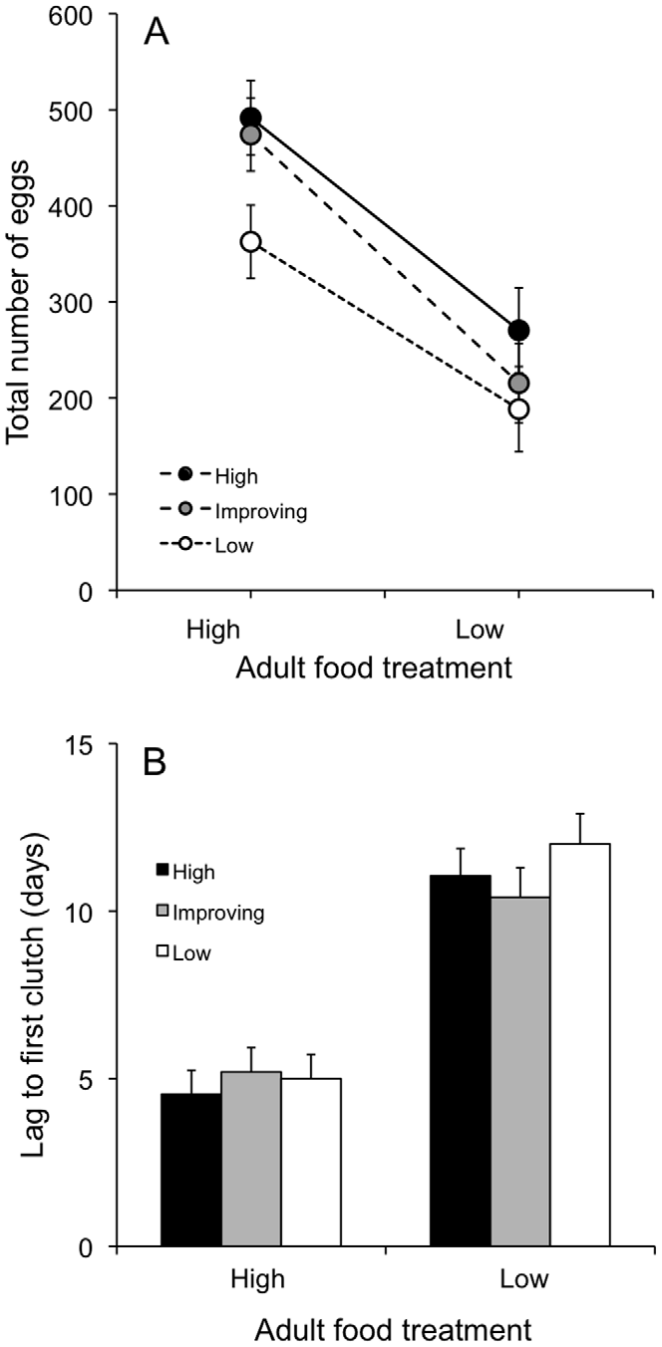
Effect of larval and adult food treatment on reproductive traits in female ladybird beetles. (A) Total number of eggs produced during the first month post-eclosion. (B) Time to first clutch. Error bars represent one standard error from the mean.

Both adult and larval food treatments had strong effects on some reproductive traits, but there was no interaction between larval and adult feeding regimes for any of these traits. There was no effect of either larval or adult food level on the time elapsed between eclosion and first mating. High adult food females began producing eggs sooner than low adult food females and produced a greater total number of eggs during the course of the experiment (Table 2, Figure 3). There was no effect of larval food treatment on the age at which oviposition began. However, larvae reared at low food produced fewer eggs over the course of the experiment than did larvae in the high and improving larval food treatments, which did not differ from one another (Tukey's *post-hoc* test *H* = *I*>*L,* Table 2, Figure 3). Because of the delay to first reproduction in the low adult food females, we also compared treatment effect on daily egg production after reproduction had commenced. The results are qualitatively similar, with low larval food and low adult food treatments having lower daily egg production and no significant interaction between larval and adult treatments (results not shown).

**Table 2:** Effect of food treatment on female reproductive traits: (a) age at first mating, (b) age at first reproduction and (c) total egg production during first month after eclosion.

	Source	df	SS	*F*	*P*
*(a) Age at mating*	Larval trt	2	0.04	0.097	0.91
	Adult trt	1	0.3	1.44	0.23
	Larval * Adult trt	2	0.87	2.06	0.13
	Error	137			
	Total	142*			
*(b) Age at first reproduction*	Larval trt	2	13.2	0.5	0.61
	Adult trt	1	1209.7	92	<0.0001
	Larval * Adult trt	2	25.53	0.65	0.52
	Error	123			
	Total	128†			
*(c) Total egg production*	Larval trt	2	272184	3.47	0.034
	Adult trt	1	1671534	42.7	<0.0001
	Larval * Adult trt	2	40935.4	0.52	0.59
	Error	137			
	Total	142*			

†Females that did not produce eggs (N = 19) were not included.

*Females that did not mate (N = 5) were not included.

## Discussion

The environment-matching hypothesis posits that plastic developmental responses to early life conditions shape the adult phenotype to fit an adult environment predicted by the larval environment. For example, developmental responses to low food should result in an adult phenotype adapted to low food in the adult stage. Here, we tested the performance of adults in low and high food environments that had developed under high, improving or low food conditions. In contrast to the predictions of the environment-matching hypothesis, there was no evidence that larvae reared in low food produced an adult phenotype that was particularly suited to a low food adult environment. Instead, adults from larvae reared in high food outperformed adults from low food, in both high and low adult food environments. Our results, and those of a recent experiment by Barrett et al. [28] on another insect having similar feeding habits in both stages, support the conventional view that resource stress during development translates into a lower lifetime fitness via its detrimental effects on body size and energy reserves, which are independent of the adult environment.

The number of experiments like ours that cross larval by adult food conditions is surprisingly low, so it remains unclear whether an adaptive phenotype matching between past and projected conditions is a common or uncommon occurrence. However, a study of the butterfly *Bicyclus anynana* [29] provides some evidence for the theory. In this species, reproductive investment declined under adult food stress for individuals reared on an *ad libitum* diet; reproductive investment of individuals reared at low food was independent of adult conditions. However, our study joins that of Barrett et al. [28] and Zajitschek et al. [30] in failing to find support for the environment-matching hypothesis.

All of our performance measures indicate that developmental responses to low larval food did not result in an adult phenotype particularly suited to a low food adult environment. Adults experiencing food restriction delayed oviposition and produced fewer eggs that those receiving abundant food, regardless of the conditions under which they were reared. Food restriction throughout the juvenile stage (the low food treatment) resulted in reduced total egg production, independent of the adult feeding treatment. Interestingly, this effect was due entirely to lower daily egg production, since there was no effect of larval diet on latency to reproduction. It may be that the risk of pre-reproductive mortality exerts a strong selective pressure on age at first reproduction, even when clutch size is reduced by resource limitation. Improving food availability prior to eclosion in the *I* treatment apparently overcame most detrimental effects of low food during the early part of development, and females in the larval *I* treatment produced a number of eggs equal to that of *H* larvae. However, despite this compensatory growth and recovery of fecundity, they still appeared to be more sensitive to adult food stress, losing as much weight the low food larvae. Simply put – good nutrition early in life results in increased adult performance in both low and high food adult environments. Interestingly, individuals that were reared at high food lost less mass in both absolute and relative terms when exposed to a period of starvation post-eclosion. This effect runs counter to the hypothesis that an excess of food may have permanent effects on metabolism that induce less efficient resource use than individuals reared on restricted diets [8]. It may be that individuals in poor condition at eclosion invested more energy in search movement; this hypothesis could be tested by assessing metabolic rates or foraging behaviour under various conditions.

Although the environment-matching hypothesis was not supported by our measurements of performance in this species, the phenotype produced by low food could, nevertheless, represent an adaptive response to food stress in environmental conditions that differ from those of our test environments. In wing polymorphic cricket and aphid species, body composition may vary predictably depending on whether the individual is wingless or flight-capable [8], [34]–[37]. The suite of traits associated with flight are strikingly similar to those observed here and elsewhere in *H. axyridis* adults that have been reared in low food as larvae. Flight-capable crickets and aphids have higher lipid content, and reduced investment in reproductive tissue, as well as a greatly reduced reproductive rate [35]–[36], implying a trade-off between reproduction and dispersal [38], [39]. These same traits are observed in *H. axyridis* adults that have been reared in low food as larvae (current study [32]). This “dispersal phenotype” induced by low larval food, may be adaptive because it allows escape from low food conditions in the adult stage. This interesting possibility will require, at least, an assessment of flight propensity and ability in adults that developed in low larval food conditions.

Our results also have relevance to the question of costs of compensatory growth [40], [41]. The substantial size disparity between larvae at day eight in the improving and high larval food treatment and was eliminated by the time of eclosion, due to a acceleration of growth rate in the improving treatment during the latter half of the larval stage when food levels had been equalized. Compensatory growth acceleration is a common phenomenon in insects and other taxa [42]–[44]. Downstream costs for accelerating growth are commonly invoked to explain the prevalence of submaximal growth in nature, although such costs have rarely been quantified [41]. The finding that larvae that underwent compensatory growth in the improving food treatment lost mass at a higher rate than those reared at high food is suggestive of a trade-off between starvation resistance and growth rate, and thus a cost of accelerated growth. Such a trade-off has been suggested earlier for this species [31] and other insects [14], [18], [45].

To summarize, when conditions experienced by developing animals predict to some degree those encountered by adults, an adaptive matching between the adult phenotype and its environment (produced by developmental plasticity) may evolve. Here, we found no evidence that the developmental response to low larval nutrition in the ladybird beetle resulted in an adult phenotype suited to low adult food. Instead, high larval nutrition produced an adult phenotype that outperformed low larval nutrition adults in both high and low adult food environments. Future studies should consider whether the lower reproductive output observed here might be offset by increased dispersal ability, thereby improving the chances of escaping poor or declining local conditions.
